# Bacteriophage origin of some minimal ATP-dependent DNA ligases: a new structure from *Burkholderia pseudomallei* with striking similarity to Chlorella virus ligase

**DOI:** 10.1038/s41598-021-98155-w

**Published:** 2021-09-21

**Authors:** Jolyn Pan, Kjersti Lian, Aili Sarre, Hanna-Kirsti S. Leiros, Adele Williamson

**Affiliations:** 1 School of Science, University of Waikato, Hamilton, 3240 New Zealand; 2 Department of Chemistry, UiT The Arctic University of Norway, 9037 Tromsø, Norway

**Keywords:** DNA, Proteins, Structural biology, Enzymes, Ligases

## Abstract

DNA ligases, the enzymes responsible for joining breaks in the phosphodiester backbone of DNA during replication and repair, vary considerably in size and structure. The smallest members of this enzyme class carry out their functions with pared-down protein scaffolds comprising only the core catalytic domains. Here we use sequence similarity network analysis of minimal DNA ligases from all biological super kingdoms, to investigate their evolutionary origins, with a particular focus on bacterial variants. This revealed that bacterial Lig C sequences cluster more closely with Eukaryote and Archaeal ligases, while bacterial Lig E sequences cluster most closely with viral sequences. Further refinement of the latter group delineates a cohesive cluster of canonical Lig E sequences that possess a leader peptide, an exclusively bacteriophage group of T7 DNA ligase homologs and a group with high similarity to the Chlorella virus DNA ligase which includes both bacterial and viral enzymes. The structure and function of the bacterially-encoded Chlorella virus homologs were further investigated by recombinantly producing and characterizing, the ATP-dependent DNA ligase from *Burkholderia pseudomallei* as well as determining its crystal structure in complex with DNA. This revealed that the enzyme has similar activity characteristics to other ATP-dependent DNA ligases, and significant structural similarity to the eukaryotic virus Chlorella virus including the positioning and DNA contacts of the binding latch region. Analysis of the genomic context of the *B. pseudomallei* ATP-dependent DNA ligase indicates it is part of a lysogenic bacteriophage present in the *B. pseudomallei* chromosome representing one likely entry point for the horizontal acquisition of ATP-dependent DNA ligases by bacteria.

## Introduction

DNA ligases are essential enzymes in DNA replication and repair, catalyzing the formation of phosphodiester bonds between adjacent 5′P and 3′OH ends in the backbone of double-stranded DNA. They are categorized as being ATP- or NAD-dependent based on the nature of the adenylate cofactor used during catalysis^1,2^. NAD-dependent DNA ligases are large, highly conserved enzymes and are primarily restricted to bacteria where they carry out the final DNA-sealing step of replication^2–4^. ATP-dependent DNA ligases by contrast are widely distributed among various taxa, and are extremely diverse in size and domain composition^2,5–8^. The roles of these different forms of ATP-dependent DNA ligases range from DNA replication to various DNA repair pathways, and for some isoforms the biological function remains unknown.

All DNA ligase enzymes possess a catalytic core comprising a nucleotidyl transferase domain (NT domain) which contains five conserved active-site motifs and is the site of catalysis, followed by an oligonucleotide binding domain (OB domain) which is responsible for engaging and positioning the DNA for ligation^9,10^. The two domains are connected by a flexible linker which allows their reorientation to encircle and engage the DNA substrate during catalysis^9^. In the majority of DNA ligases, this catalytic core is appended N- or C-terminally by additional modules which enhance ligation efficiency, or possess autonomous enzymatic activities. However, a small sub-set of DNA ligases lack additional globular domains, instead using extended loops or positively-charged binding motifs to engage their DNA substrates^11,12^, or relying on recruitment by additional binding partners^13,14^. These minimal DNA ligases, all within the ATP-dependent sub-class, include viral ligases from Chlorella virus and T7 bacteriophage which were described in foundational structure–function studies of DNA ligases^12,15^. More recently, the minimal DNA ligases of bacteria have received attention including publication of several new structures with and without DNA substrate bound^11,16^. To date two types of minimal bacterial DNA ligase have been biochemically characterized: ‘Lig C’ (i.e. ligases with domains PF01068 and PF04679) which interacts with multiple base excision repair enzymes^13^, and ‘Lig E’ (PF01068 and PF14743) which does not require a binding partner for activity and possesses a predicted periplasmic leader sequence at its N terminus^17^. Lig C is involved in stationary-phase base excision repair in actinobacteria^13^, while the biological function of Lig E is not known, although a role in DNA uptake has been suggested^18,19^.

In our recently-published study^2^, sequence similarity networks (SSNs) were used to survey the sequence diversity of DNA ligases among all kingdoms of life; however, that work specifically excluded sequences less than 300 amino acids as these did not form cohesive clusters with larger homologs. SSNs are an alignment-based method that can be used to determine relationships between groups of sequences where construction of phylogenetic trees is unsuitable either because the number of homologs is too large to be feasible, or because sequence diversity leads to poor branch support. Although not as robust as phylogenetic trees for constructing evolutionary histories, these networks can provide considerable insight into the diversity and similarity of proteins within a given family^20,21^. The present study focuses on minimal DNA ligases, as defined above, such as Chlorella virus and Lig E-type ligases which lack appending domains. The purpose is to determine (1) what sequence and potential structural diversity is present in these variants and (2) potential evolutionary trajectories for the differential distribution of these genes among organisms.

## Results

### Sequence similarity network analysis

**Figure 1:**
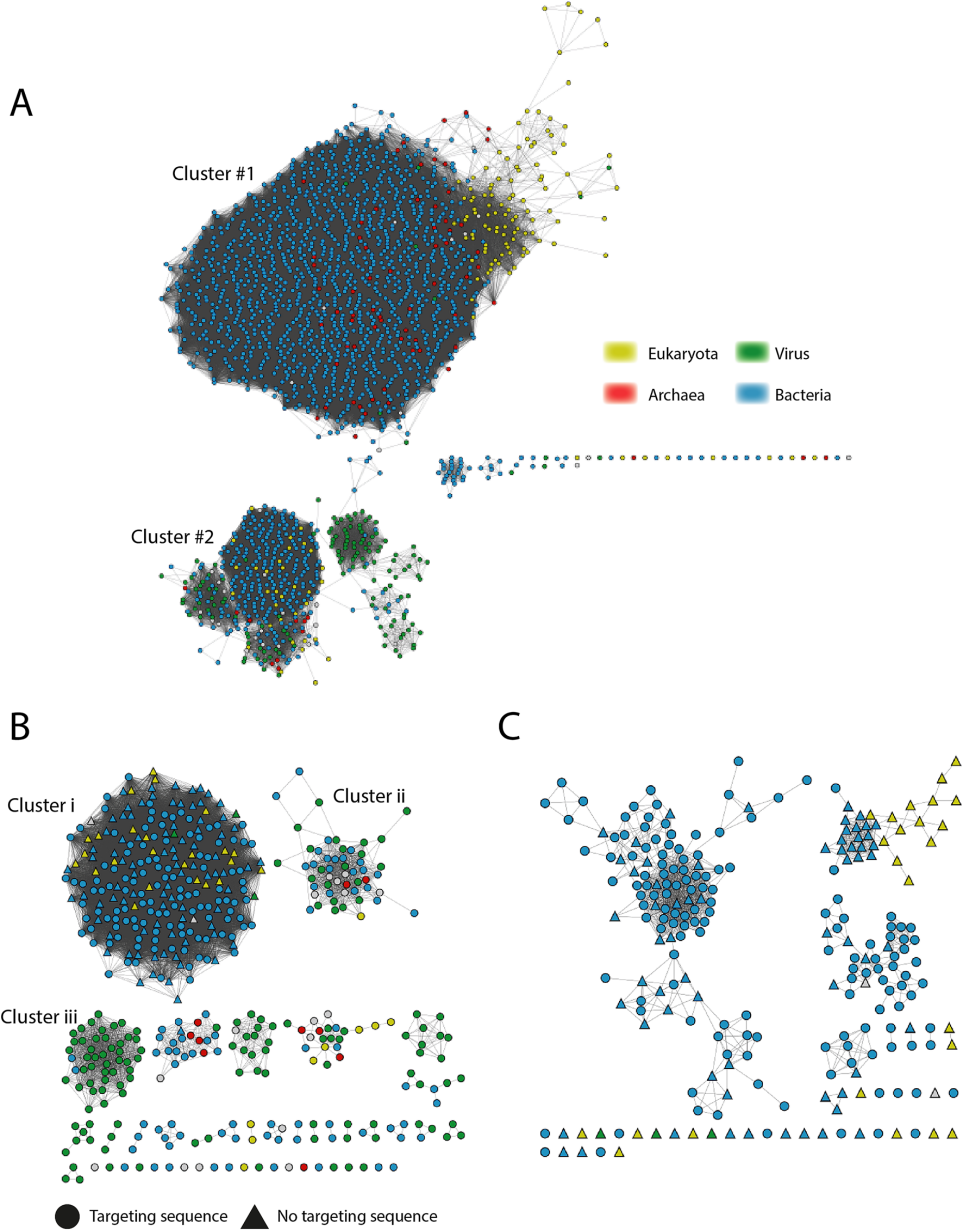
Repnode Sequence Similarity Network of DNA ligases 250–370 amino acids long. (**A**) Clusters of all sequences at 22% edge cutoff. (**B**) Cluster #2 refined to 37% edge cutoff. (**C**) Cluster i refined to 52% edge cutoff. Nodes throughout are coloured by repnode taxa as indicated in the key (Eukaryotes, yellow; Archaea, red; Viruses, green; Bacteria, blue). For panels (**B**,**C**), the presence or absence of a leader sequence is indicated by node symbol shape (circle and triangle, respectively).

To survey the sequence diversity and distribution of minimal DNA ligases, a SSN of DNA ligases between 250 and 370 residues was constructed, (summarized in Table 1) and the constituent sequences were categorized into ligase types based on their Pfam domain composition. The initial network included a total of 17,011 sequences represented by 2020 nodes and formed two major clusters (Fig. 1A). Cluster #1 includes predominantly bacterial Lig C sequences as well as partial sequences of bacterial Lig B, Lig D and replicative Eukaryotic ligases. Cluster #2 includes bacterial Lig E in addition to viral representatives both of Chlorella virus and T7 types and a small number of eukaryotic sequences. Both clusters include a considerable portion of sequences annotated as ‘NT-only’ which appear to be partial sequences.

**Table 1:** Features of Sequence Similarity Networks constructed with DNA ligases 250–370 amino acids long.

Network name	Cutoff (% identity)	Cluster	Number of repnodes	Number of edges
All minimal ligases	22	#1 (Lig C, partials)	1472	356,340
		#2 (Lig E, partials, viral)	481	29,808
Cluster # 2	38	i (Lig E)	237	18,481
		ii (ChlV-like)	52	568
		iii (T7-like)	45	795
Cluster i	52	a (Beta-, Gammaproteobacteria)	112	752
		b (Epsilonproteobacteria	38	107
		c (Deltaproteobacteria)	34	152
		d (Epsilonproteobacteria)	8	27

At the 20% identity threshold, several groupings within Cluster #2 are associated by a relatively small number of edges between 30 and 40%, thus to further refine associations between the sequences in Cluster #2 a new sub-network was generated with an edge threshold of 37% identity (Fig. 1B). The resulting new clusters define three major groups: Cluster i with mainly canonical proteobacterial Lig E proteins including characterized representatives from *Alteromonas mediterranea*, *Psychromonas* sp. strain SP041, *Aliivibrio salmonicida, Neisseria meningitidis* and *Haemophilus influenzae*; Cluster ii, comprising Chlorella virus-like ligases from both bacteria and viruses; Cluster iii, with T7-like ligases which were almost entirely Podoviridae bacteriophage. Smaller clusters and singleton sequences include predominantly bacterial candidate phyla and metagenomic sequences, as well as additional bacteriophage sequences.

As many bacterial Lig E sequences are predicted to possess periplasmic targeting sequences, Signal P was used to annotate the representative node (repnode) sequences in the Cluster #2 sub-network. Signal sequences were indicated only for members of the canonical Lig E cluster (Cluster i), with 144 of the 208 bacterial repnode sequences (69%) having such a prediction. Additional refining of this cluster to an edge threshold of 52% identity (Fig. 1C) revealed that the majority of leader-less sequences are from the Deltaproteobacteria Myxococcales and form a cohesive cluster (Cluster c). Intriguingly, these group together with a small number of eukaryotic, mainly fungal, sequences in this network. Examination of the genome sequences of two bacterial representatives from this leader-less group, *Myxococcus macrosporus* and *Cystobacter fuscus* did not find any evidence of alternative start-sites upstream of the coding sequence, suggesting that the N-terminal region is correctly annotated.

Three groupings within the 52% identity threshold include the majority of remaining sequences, all of which are from Proteobacteria. The larger of these (Cluster a, 112 nodes) is almost entirely from Beta- and Gammaproteobacteria, while the two smaller groups (Cluster b, 38 nodes and Cluster d, 8 nodes) are mainly Epsilonproteobacteria, including a large number of *Campylobacter* isolates.

### Analysis of the Lig E cluster

**Figure 2:**
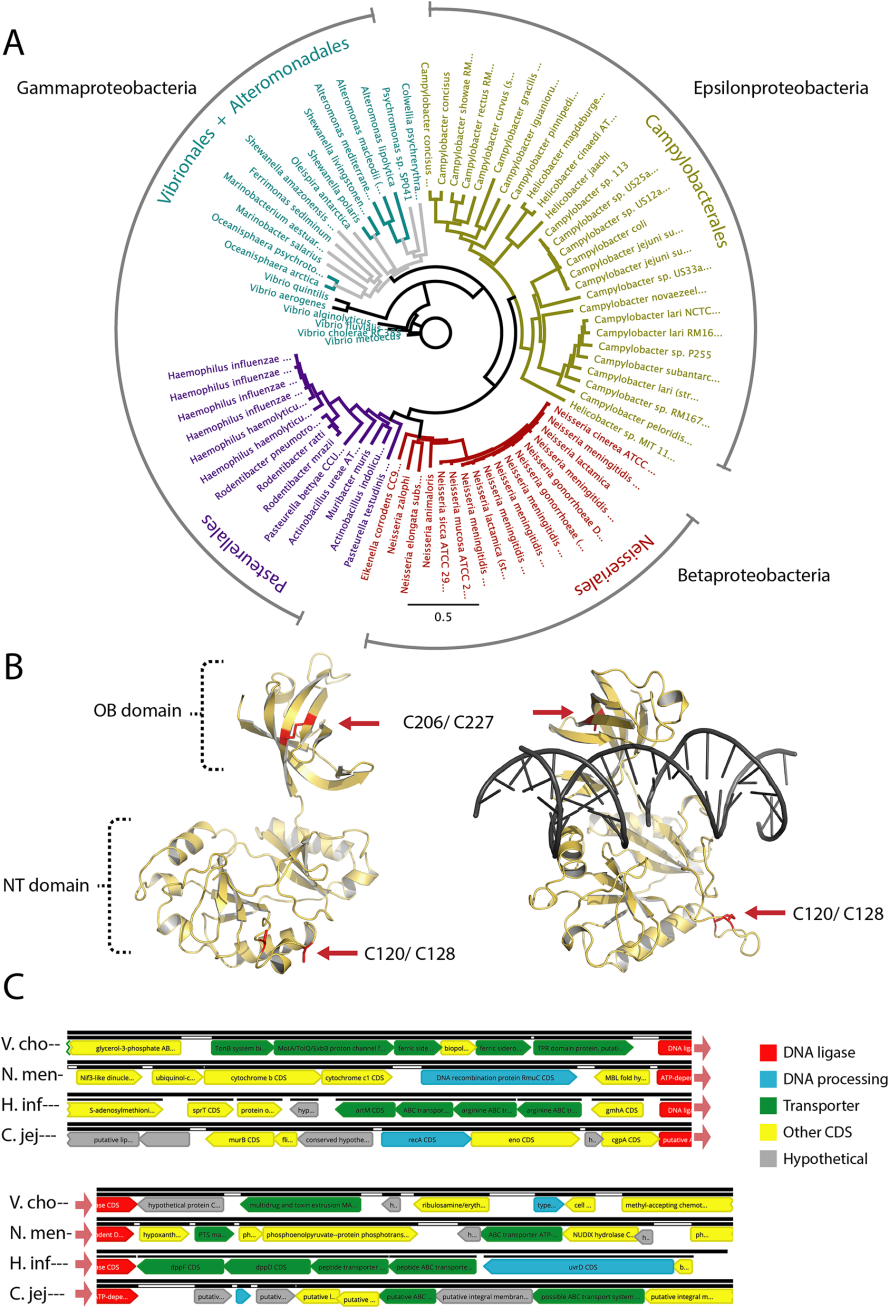
(**A**) Phylogenetic analysis of bacterial Lig E sequences coloured by Order. Solid-coloured branches indicate bootstrap values greater than 50% consensus support. Protein identifiers for ligases are given in Supplementary Table 1. (**B**) Lig E from Campylobacter jejuni modeled in open DNA-free (left) and closed DNA-bound (right) conformations. Potential disulfide bonds in the OB and NT domains are indicated in red. (**C**) Genomic context of Lig E from pathogenic representatives of different bacterial Orders. Species names are abbreviated as: *V*. *cho, Vibrio cholera:* *N.* *men*, *Neisseria meningitidis*; *H. infl*,* Haemophilus influenzae*; *C. jej*, *Campylobacter jejuni*. Genes are coloured according to the function annotated in the genomic sequence as DNA processing (blue), transporter (green), other known function unrelated to transport or DNA (yellow) or unknown function (grey). The DNA ligase is coloured red for orientation.

The refined SSN (52% identity) indicates that differences in the Lig E sequences correlate with taxonomic groupings, which is consistent with the previous finding that most proteobacterial Lig E enzymes are vertically inherited^19^. Phylogenetic analysis of a sub-set of Lig E sequences from the SSN confirms they form distinct order-level clades with one or more pathogenic representatives in the group (Fig. 2A). The Lig E of *H. influenzae* along with other sequences from Pasteurellaceae is placed closer to the Betaproteobacterial Neisseriales Lig Es, which as noted previously, may be evidence for a more recent gene acquisition in this group^19^.

As both available structures of Lig E are from marine Gammaproteobacteria, we used a homology modelling approach to analyze structural differences between Lig E from pathogenic Gammaproteobacteria (*Vibrio cholerae* and *Haemophilus influenzae;* hereafter Vcho-lig and Hinf-lig, respectively)*,* Betaproteobacteria (*Neisseria meningitidis* hereafter Nmen-lig), and Epsilonproteobacteria (*Campylobacter jejuni*, hereafter Cjej-lig). The highest scoring models for all four sequences were the previous DNA-free Lig E structure from *Psychromonas* SP041 (hereafter Psy-lig) and the DNA-bound structure from *Alteromonas mediterranea* (hereafter Ame-lig) (Table 2). Sequences from Epsilonproteobacteria and Betaproteobacteria classes as well as the Pasteurellaceae order have a highly conserved pair of cysteines in the OB domain (Fig. 2B, Supplementary Fig. 1). In Hinf-lig, Nmen-lig and Cjej-lig, these are modeled in close proximity and are predicted to form disulfide bonds in the DNA-free state both in structural models, and by the disulfide prediction server DiANNA^22^. A second cysteine pair in a conserved NT-domain loop in many *Campylobacter* representatives (Cjej-lig C120 and C128) is predicted to form a disulfide bond in the DNA-bound state only.

**Table 2:** Sequence identity and quality scores of homology models for pathogen Lig Es based on structurally-characterized representatives in the DNA-bound (Ame-lig) and DNA-free (Psy-lig) conformations.

	Ame-lig % ID (Qmean)	Psy-lig % ID (Qmean)	Cys pairs predicted by DiANNA
*V. cholera*	41.86 (− 0.92)	44.14 (− 0.10)	NA
*N. meningitides*	35.57 (− 0.57)	40.48 (0.00)	C200–C221
*H. influenzae*	35.97 (− 1.52)	37.85(− 0.68)	C196–C217
*C. jejuni*	35.74 (− 1.56)	36.58 (− 0.82)	C120–C128
			C206–C227

Disulfide connectivity is predicted by highest-scoring cysteine pairs from DiANNA.

Many other bacterial ATP-dependent DNA ligases are found in close genetic proximity to genes encoding proteins with which they interact^13,23^, therefore we investigated conservation of genes adjacent to Lig E. There was no synteny in gene organization between these pathogens, nor were the functions of adjacent genes generally conserved (Fig. 2C, Supplementary Table 3). Both *V. cholera* and *H. influenzae* DNA ligases are surrounded by genes encoding membrane transporters including siderophore transporters, efflux pumps, amino acid and peptide transporters, while the ligase of *N. meningitides* is flanked by enzymes involved in nucleotide biosynthesis and energy utilization. The DNA ligase of *C. jejuni* is surrounded by other putative periplasmic-coding sequences, and is upstream of a tRNA operon. In all four pathogen genomes, another predicted DNA-processing enzyme was encoded within five genes of the DNA ligase. These include proteins with functions in recombination (*N. meningitides* RmuC and *C. jejuni* RecA), nucleotide excision (*H. influenzae* UvrD) and replication (*V. cholera* type II topoisomerase); however, none points to a consistent pathway in which Lig E might function.

Analyses at lower taxonomic levels indicate essentially no synteny outside individual species for *Campylobacter* (Supplementary Fig. 2), while only the directly adjacent genes are preserved for *Vibrios* (hypothetical lipoprotein, MATE efflux transporter) and *Neisseria* (metallohydrolase fold protein) (Supplementary Figs. 3, 4). More extensive synteny was detected in *Haemophilus* species possessing Lig E, with the series of arginine transporters and d-sedoheptulose 7-phosphate isomerase genes from *H. influenzae* being present in H. *aegyptius and H. parainfluenzae* (Supplementary Fig. 5)*.* However, as Lig E is absent from many other *Haemophilus* representatives, notably all strains of *H. ducyeri*, this conservation likely reflects the narrow distribution of Lig E among these genera rather than a functional association.

### Structure and activity of minimal DNA ligase from *Burkholderia pseudomallei*

In addition to the Lig E-containing Cluster i, the refined SSN at a 37% edge level defined a second major group, Cluster ii. This cluster contains a large number of bacterial representatives (22 nodes), together with viral representatives (21 nodes), including the well-characterized Chlorella virus ligase (hereafter ChlV-lig). All DNA ligase sequences within this group have the latch insert in the OB domain that was shown to be necessary for substrate engagement and high levels of activity in ChlV-lig^12,24^, and neither bacterial sequences, nor those annotated as being from bacteriophage possess predicted leader sequences. These features, in addition to their segregation from Lig E in the SSN at higher edge thresholds indicate these bacterially-encoded ChlV-lig homologs have different biological functions. Among the bacterial representatives with Chlorella virus-like ligases are several species of *Burkholderia*, some of which cause serious human diseases such as *B. pseudomallei,* the causative agent of melioidosis^25^. To gain insight into the structure and function of ligases from this group, we recombinantly expressed and characterized the minimal-type ATP-dependent DNA ligase from *B. pseudomallei* (hereafter Bsp-lig). Results of purification, are given supplementary file 6.

**Figure 3:**
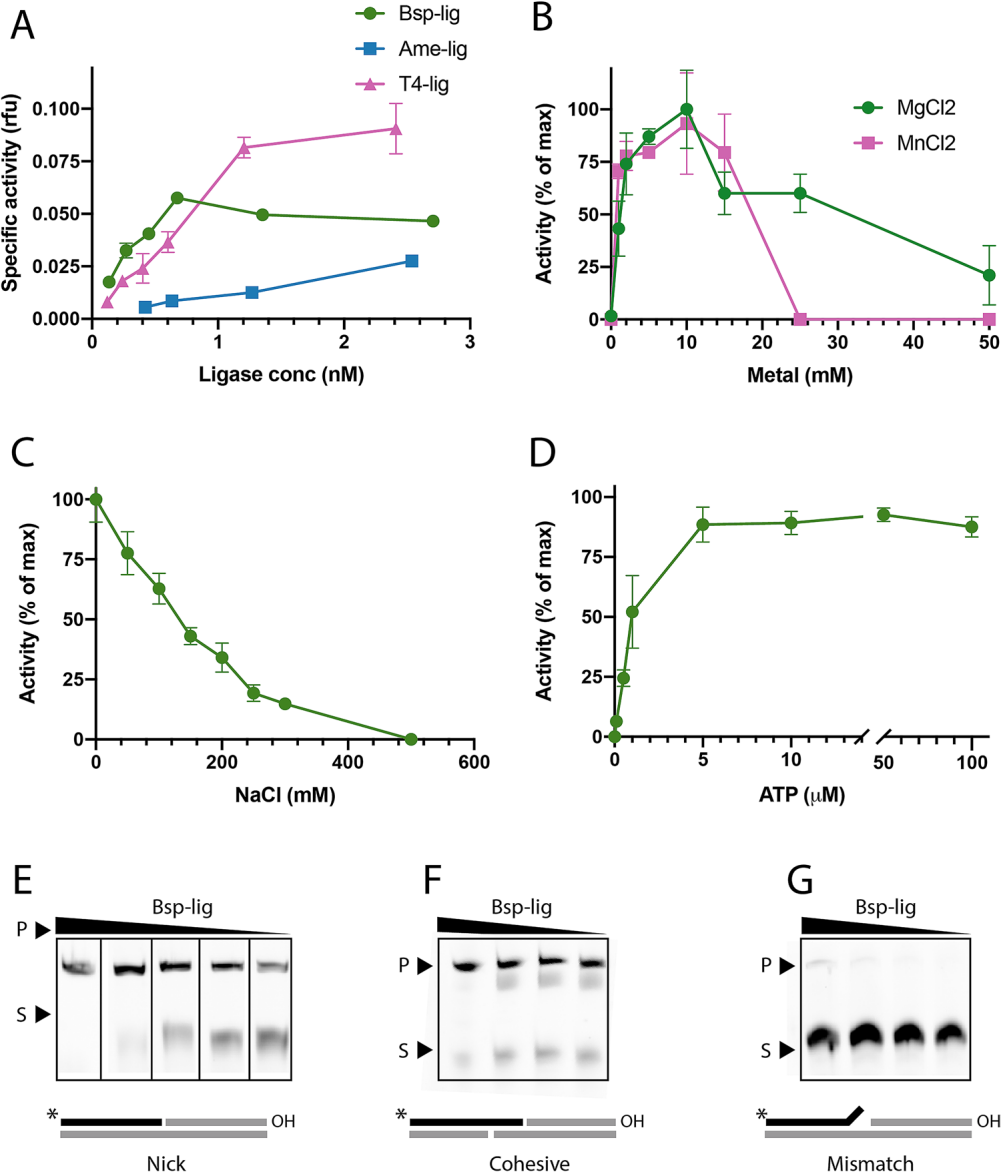
DNA ligase activity of Bsp-lig. Measured by molecular beacon assay (**A**-**D**) or urea-PAGE (**E**–**G**). (**A**) Specific activity of Bsp-lig (green circle) relative to Ame-lig (blue square) and T4 DNA ligase (purple triangle). (**B**) Relative activity of Bsp-lig with MgCl_2_ as the divalent cation (green circle) or MnCl_2_ (purple square). (**C**) Effect of NaCl on Bsp-lig activity). (**D**) Effect of ATP concentration on Bsp-Lig activity. (**E**) Bsp-lig activity with nicked substrate (Bsp-lig concentrations 20 nM, 10 nM, 5.0 nM, 2.5 nM, 1.8 nM). (**F**) Bsp-lig activity with cohesive-end substrate (Bsp-lig concentrations 550 nM, 55 nM, 20 nM, 10 nM). (**G**) Bsp-lig activity with mismatch substrate (Bsp-lig concentrations 550 nM, 55 nM, 20 nM, 10 nM). Uncropped gels are shown in Supplementary Fig. 7.

Bsp-lig is extremely effective in nick-sealing assays with higher specific activity than the canonical bacterial Lig E Ame-lig, and similar activity to T4 DNA ligase in the lower enzyme concentration range (Fig. 3A,E). It is able to use both MgCl_2_ and MnCl_2_ as divalent cation cofactors in the range of 1.0–10.0 mM. However, it is completely inhibited by MnCl_2_ at 25 mM, whereas 50% of specific activity is retained with MgCl_2_ (Fig. 3B). As with many DNA ligases^11,17^, Bsp-lig is inhibited by salt in a linear fashion, retaining only approximately 25% activity at 200 mM NaCl (Fig. 3C). It has a minimal ATP concentration of 5 M$\mu$ for optimal activity with no significant inhibition up to 100 M$\mu$ (Fig. 3D). Bsp-lig is able to seal cohesive-end double-strand breaks and, to a lesser degree, mismatched nicks (Fig. 3F,G); however, it has no detectable activity on blunt-ended double-strand breaks or single-break substrates with a gap at the ligation site (data not shown).

**Figure 4:**
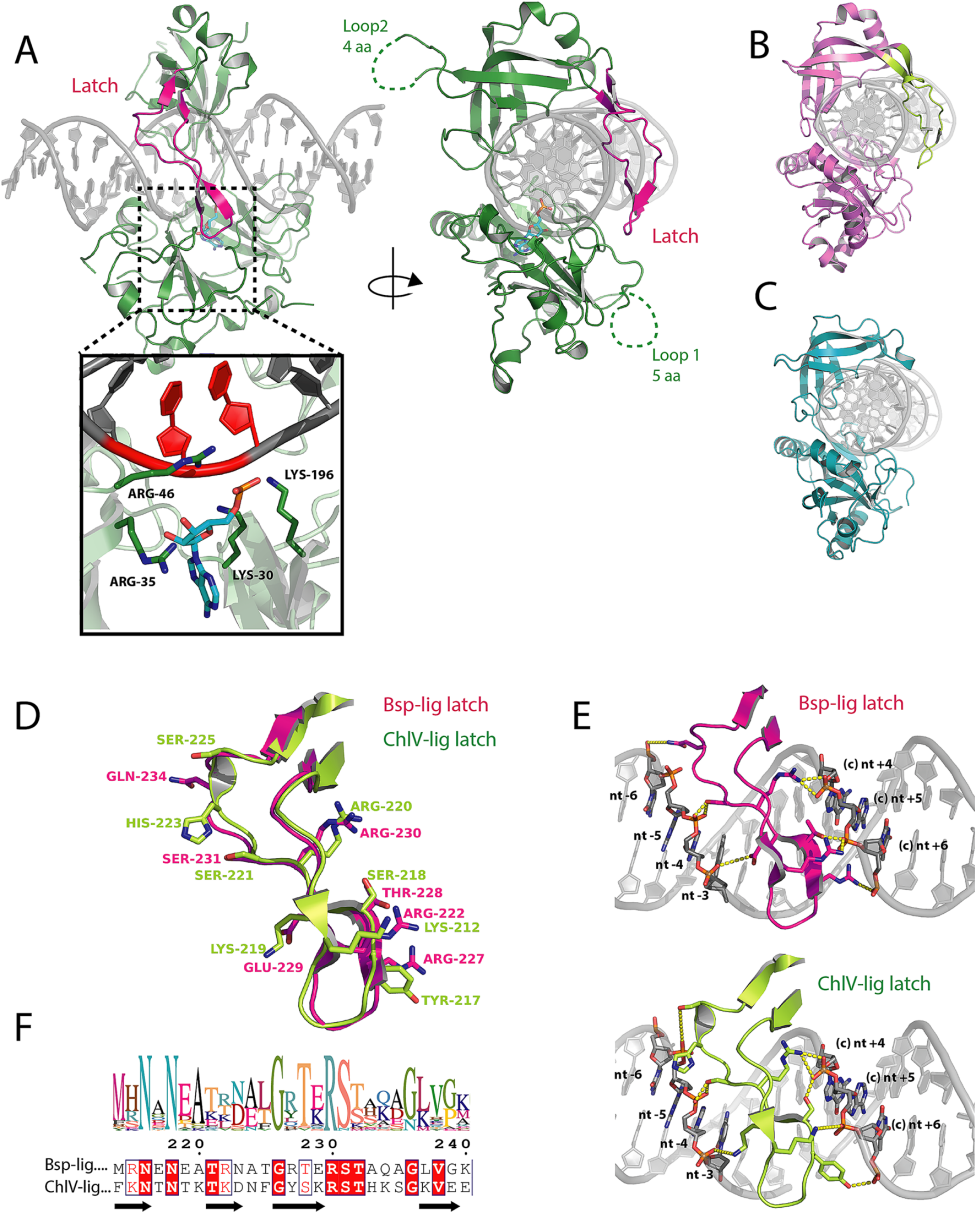
Structure of Bsp-lig bound to post-ligation product DNA. (**A**) Overall structure with latch region highlighted in magenta. Inset shows Bsp-lig active site with ligated nucleotides in DNA substrate indicated in red. AMP in cyan and key active site residues of Bsp-lig as green sticks. (**B**) Structure of ChlV-lig bound to DNA with latch highlighted in lime. (**C**) Structure of Ame-lig bound to DNA. (**D**) Superposition of latch regions from Bsp-lig (magenta) and ChlV-lig (lime) with key DNA-interacting residues shown as sticks. (**E**) Detailed view of interactions of Bsp-lig latch (magenta, top) and ChlV-lig latch (lime, lower) with DNA. DNA nucleotides and protein side-chains involved in interactions are shown as sticks, hydrogen bonds and salt bridges are indicated with yellow dashed lines. (**F**) Sequence logo of latch region for 28 Bsp-lig homologs (top) and alignment between latches of Bsp-lig and ChlV-lig (lower) with fully-conserved residues highlighted in red and synonymous differences indicated in red text.

The new 2.45 Å resolution crystal structure of Bsp-lig in complex with DNA to shows the classical conformation where the ligase encircles the double-stranded substrate. The concave face of the OB-domain positioned in the minor groove and the ligated strand positioned over the active site of the NT domain (Fig. 4A). Covalently adenylated Bsp-lig was co-crystalized in complex with nicked DNA substrate, however the electron density maps show that a significant proportion has been ligated by the enzyme, and the linear sealed form is modeled in the active site of the resulting structure (Fig. 4A, inset). Covalently-bound AMP was refined in the active site of the structure with an occupancy of 0.6, however only two of the three phosphate atoms could be accurately placed and the third phosphate was omitted from the model (Supplementary Fig. 8B).

The latch region of Bsp-lig is positioned in the major groove across the ligation site and its complementary nucleotides, making kissing contacts with the NT domain. This arrangement is overall similar to the conformation of ChlV-lig bound to DNA (Fig. 4B), and differs from the bacterial Lig E which lack the latch region (Fig. 4C). Comparison of the latch regions of Bsp-lig and ChlV-lig show a similar structural arrangement (Fig. 4D) and protein-DNA contacts (Fig. 4E). Multiple alignment of Bsp-lig homologs shows the most highly conserved positions are the pair of asparagines (Bsp-lig Asn216 and Asn219) in the N-terminal beta strand, a glycine residue at the beta hairpin in the tip of the latch (Bsp-lig Gly 226) and the T(E/K)RS motif at the end of the third β-strand (Bsp-lig Thr228-S231) (Fig. 4F). Of these positions, only the latter motif makes direct sidechain contacts with the DNA via Thr 228 to T7 of the complement strand +6 nucleotides from the nick site (nt+6), Glu229 to nucleotide C29 on the nicked strand in the −3 position (nt−3), Arg 230 to nucleotide G9 in the +4 position from the nick on the complement strand (nt+4) and Ser 231 to nucleotide A26 in the −4 position from the nick (nt−4). Comparison of latch structures show that other key contacts are functionally substituted between Bsp-lig and ChlV-lig, despite the lack of consensus in other sequences. For example nucleotide A8 of the complement strand +5 from the nick (nt+5) forms a bond with Arg 222 of Bsp-lig and Lys212 of ChlV-lig, but these may be substituted for threonine or leucine in other species; nucleotide C7 in the +6 position from the nick on the complement strand (nt+6) forms a salt bridge with Arg 227 of Bsp-lig, but this is replaced with a hydrogen bond from Tyr 217 of ChlV-lig, and this position can also be leucine or glutamine.

The major differences between the structures of Bsp-lig and ChlV-lig are two partially unstructured loop regions in Bsp-lig which are absent in ChlV-lig (Fig. 4A, (Supplementary Fig. 8A). The first of these, Pro109-Met119 in the NT domain (loop1), has five residues with no electron density (Ile112-Ser116) and is part of a highly variable region in the sequence alignment between 11 residues (Bsp-lig) and 6 residues (ChlV-lig). The second unstructured loop region in the OB domain Phe251-Gly264 (loop 2) has 4 unresolved residues (Arg254-Gly257) and is likewise in a poorly conserved region that varies between 14 (Bsp-lig) and 2 (ChlV-lig) residues in length. Neither of these loops are optimally positioned for DNA interaction, and their lack of density in the Bsp-lig structure suggests they are not involved in substrate binding.n.

### Phage origin of Bsp-lig and its homologs in bacterial genomes

**Figure 5:**
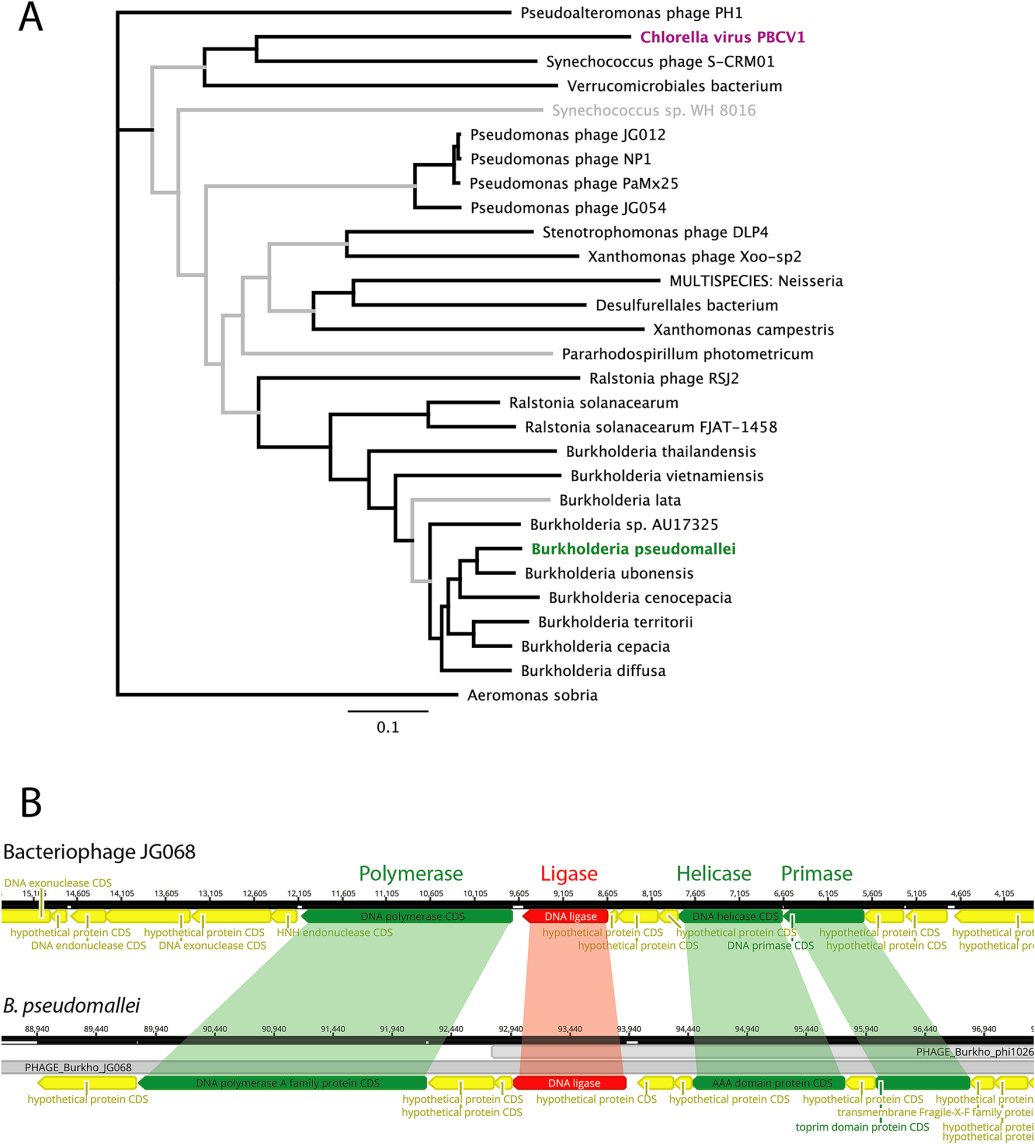
(**A**) Neighbor-Joining consensus tree for amino acid sequences of Bsp-lig homologs with Bsp-lig highlighted in green and ChlV-lig in magenta. Branches with > 50% consensus support are boldened in black. Sequence identifiers for ligases are given in Supplementary Table 2. (**B**) Genomic context of Bsp-lig and its homolog in bacteriophage JG068. Adjacent DNA processing genes with high similarity are highlighted in green. Bacteriophage regions predicted in *B. pseudomallei* are indicated in grey.

A phylogenetic tree built from a sub-set of Bsp-lig homologs from Cluster ii shows that the genomically-encoded bacterial ligases and those annotated as bacteriophage do not form distinct clades, suggesting these particular bacterial DNA ligases may be part of lysogenetic bacteriophage residing within their genomes (Fig. 5A). Prediction of bacteriophage regions within the Bsp-lig contig of *B. pseudomallei* indicated two overlapping regions of likely phage origin with Bsp-lig being located within both (Table 3). The complete region includes essential genes for phage replication such as Terminase, Head, Tail and Capsid genes, while the incomplete region includes a number of nucleic acid-processing enzymes including polymerases (both DNA and RNA dependent) and nucleases.

**Table 3:** Phage prediction for the region of B. pseudomallei BES encoding Bsp-lig.

Region	Region length (kb)	Position	Completeness (score)	# proteins (Phage (total))	Top prediction (identifier)
1	32.1	70,946–103,098	Incomplete (50)	28 (49)	PHAGE_Burkho_JG068 (NC_022916)
2	33	92,786–125,822	Intact (140)	20 (24)	PHAGE_Burkho_phi1026b (NC_005284)

The genome of bacteriophage phi1026b, the top prediction for the complete phage region, does not contain a DNA ligase gene. However, phage JG068, the top hit for the incomplete phage, possesses a DNA ligase with 34% identity and 55% similarity (Blosum62) to Bsp-lig at the amino acid level, and has the predicted latch region seen in Bsp-lig and ChlV-lig. In both *B. pseudomallei* and JG068 the DNA ligase is flanked by DNA processing enzymes including a polymerase, helicase and primase (Fig. 5B). Pairwise comparison of these genes shows significant similarity at both the amino acid and nucleotide level which strongly indicates that Bsp-lig is indeed part of a lysogenic bacteriophage, similar to the obligately lytic podovirus phage JG068 (Table 4).

**Table 4:** Homology between genes adjacent to Bsp-lig in *B. pseudomallei* BES and those in bacteriophage JG068.

Protein	Gene identifier		Nucleotide identity (%)	Amino acid identity (amino acid similarity) (%)
	*B. pseudomallei*	phage JG068		
DNA polymerase	KGD55320.1	YP_008853857.1	64	61 (77)
Helicase	KGD55327.1	YP_008853852.1	67	60 (85)
Primase	KGD55358.1	YP_008853851.1	57	44 (71)

## Discussion

Analysis of minimal ATP-dependent DNA ligases by sequence similarity networks revealed that the vast majority of these enzymes are bacterial, despite being less known than the well-characterized viral representatives from Chlorella virus and T7 bacteriophage^12,15^. A similar situation was seen with the larger ATP-dependent DNA ligases where bacterial sequences comprised more than 60% of the dataset^2^. This is despite the non-essential role and non-ubiquitous distribution of ATP-dependent DNA ligases in bacteria^3,6,19^, and can in part be attributed to the predominance of bacterial sequences in the databases relative to eukaryotes, viruses and archaea^2^.

The formation of two major clusters within the initial SSN which grouped Lig C alone and Lig E with viral ligases is consistent with previous phylogenomic analyses on a smaller set of 65 DNA ligase sequences^19^. That previous study, which focused on Bacteria, found that the Lig E-type ligases had structural features and distributions distinct from all other bacterial ATP-dependent DNA ligases, grouping closer to bacteriophage enzymes^19^.

In the present study, further refinement of SSN clusters through higher stringency thresholds resolved the canonical Lig E-type ligases as a cohesive group separate from bacteriophage and other viral enzymes. In addition, of > 17,000 sequences from 42 phyla in the main SSN, the 208 bacterial sequences Lig E subnetwork (Cluster #2, i, a) was essentially all proteobacterial with < 10 being from other phyla (Planctomycetes and Verrucomicrobia). This is consistent with the previous finding that the Lig E-type ATP-dependent DNA ligases are restricted to Proteobacteria^19^. Together with the majority prediction of a leader peptide in Lig Es of our expanded dataset and phylogenetic evidence of vertical inheritance, this supports the notion of this as a class of proteobacteria-specific enzymes with a distinct biological function^19^. Analysis of Lig E-encoding regions from numerous bacterial chromosomes did not reveal any operon-encoded enzymes that might participate in multi-step pathways as is seen for the bacterial DNA ligases which participate in base excision-repair (Lig C) and non-homologous end joining repair (Lig D)^8,13^. Likewise, prediction of bacteriophage regions in these genomes did not indicate that Lig E is part of a lysogenic bacteriophage, either complete or partial. Although the common clustering of Lig E and other small ligases with phage at lower stringency-levels of the SSN provides plausible support for a phage-origin, it did not identify a ‘missing link’ where such a gene was horizontally transferred. The lack of common synteny in the Lig E -encoding region means elucidation of any interaction partners must await cellular-based experiments for clues to its biological function and any relevant pathways.

In contrast, Cluster #2 ligases outside of the Lig E group which were annotated as bacterially-encoded in UniProt had clear links to a bacteriophage origin. These include high sequence similarity to bacteriophage genes and prediction as components of lysogenic bacteriophage residing within bacterial chromosomes. Characterization of one such enzyme, the ATP-dependent DNA ligase of *B. pseudomallei* Bsp-lig revealed it has similar features to the Chlorella virus DNA ligase ChlV-Lig including the presence of a 27 residue latch extension within the OB domain that engaged the DNA substrate in the bound form^12^. Despite having only 9 fully-conserved and three partially-conserved positions between the two proteins, functionally equivalent contacts are made between the DNA and the latch for both enzymes and this structural similarity is reflected in its enzymatic properties which include its preference for singly nicked or cohesive-ended substrates and poor activity on gapped DNA duplexes^26,27^. Comparison of the Bsp-lig and ChlV-lig DNA-bound structures^12^ has highlighted two loop regions in the Bsp-lig structure, which vary in both length and amino acid composition in other bacterial and phage proteins, however both are positioned away from the DNA substrate, suggesting they do not participate in binding.

Prediction that Bsp-lig and related ligases are of bacteriophage origin suggests that they have been recently acquired through phage infection and, unlike Lig E, may not be vertically inherited or of biological significance to the encoding bacteria.

In conclusion, this study highlights the ever-expanding diversity and complexity of DNA ligases encoded within bacterial genomes, which will continue to grow with the exponential increase in available sequences. The exact source of horizontal transfer of the Lig E-type DNA ligases into proteobacteria remains unknown; however this study provides further evidence of a viral origin for this gene. The restricted distribution of Lig E to proteobacteria, combined with a near-ubiquitous prediction for a periplasmic export signal provides further support to an extracellular biological function for Lig E while a lack of synteny with surrounding genes does not immediately indicate any interaction partners in this function. The identification and characterization of a bacterial DNA ligase with high similarity to the Chlorella virus enzyme indicates that this configuration of DNA binding via an OB loop latch is not restricted to close phylogenetic relatives of the latter protein, but may represent a more widespread mode of interaction. Identification of Bsp-lig as residing within a lysogenic phage in the *B. pseudomallei* chromosome highlights the necessity of interrogating the genomic context of such enzymes before ascribing biological function, and also suggests a mechanism for horizontal transfer of minimal ligases into bacterial genomes.

## Methods

### SSN construction and annotation

The initial SSN was constructed from sequences in InterPro90 (https://www.ebi.ac.uk/interpro/) using the EFI-EST server (https://efi.igb.illinois.edu/efi-est/)^28^ with the families option using the NT domain of ATP-dependent DNA ligase (PF01068, *DNA_ligase_A_M*) as input and sequence length of 250–370 residues. An E-value of 5 was used for initial SSN calculation, and the threshold was set to 20 (corresponding to 21% sequence identity) for the final network. Cytoscape v3.2.8 (https://cytoscape.org/) was used to visualize and further process the network, and to reduce network size, the repnode 40 network where all sequences with greater than 40% identity over 80% of length are represented as a single node was used. DNA ligase type was assigned on the basis of the complement of Pfam domains as described previously^2^; to summarize, OB domain PF04679 (bacterial Lig C; partial sequences of bacterial Lig B and Lig D; partial sequences of eukaryotic Ligase I and III), OB domain PF14743 (Bacterial Lig E; Chlorella virus-like ligases; partial fungal sequences of unknown function) and PF17879 (bacteriophage T7-like ligases). Partial hits were assigned on the basis that appending domains found in larger ligases were detected. Signal sequences were predicted using the SignalP server (http://www.cbs.dtu.dk/services/SignalP/)^29^ in both Gram negative and Gram positive mode.

### Genome context, Synteny analysis and bacteriophage prediction

The following genome sequences were downloaded from the NCBI (https://www.ncbi.nlm.nih.gov/) for use in analysis: *Burkholderia pseudomallei* strain BES contig 542 (JPHA01000251.1); Burkholderia phage (JG068 NC_022916); *Campylobacter jejuni* subsp. jejuni NCTC 11168 (AL111168); *Haemophilus influenzae* strain Hi375, (CP009610); *Neisseria meningitidis* strain 11-7 (CP021520); *Vibrio cholerae* MS6 (AP014524). Genomes were visualized using Geneious prime software version 1.3, and bacteriophage prediction used the PHASTER web server (https://phaster.ca/)^30^ Synteny analysis was conducted using the SynTax server (https://archaea.i2bc.paris-saclay.fr/SyntTax/)^31^.

### Sequence alignment and phylogeny analysis

Sequences used to construct phylogenetic trees of Lig E DNA ligases and Chlorella virus-like ligases are given in Supplementary Tables 1 and 2. To generate multiple alignments, sequences were aligned in Geneious software (Geneious Prime^®^ 2019.version 1.3, www.geneious.com) using the ClustlW version 2.1 plugin (Blosum 62 matrix, gap open cost 10, gap extend cost 0.1). For Lig E sequences, N terminus of sequences in the initial alignment was trimmed by 30 amino acids to remove the predicted leader sequence, and sequences were then re-aligned. Phylogenetic trees used the Geneious tree builder to construct Neighbour-joining trees from these alignments using the Jukes-Cantor distance model and 500 bootstrap replicates.

### Homology modelling

Amino acid sequences of Lig E were extracted from the genomes of *V. cholera* (BAP02982.1) *N. meningitides* (QEN75767.1) *H. influenzae* (AIT68169.1) and *C. jejuni* (CAL35765.1) and submitted to the Swissmodel server^32^. Best scoring templates for all sequences were Lig E from *Pychromonas* SP041 (PDB ID: 4d05) and *A. mediterranea* (PDB ID: 6gdr), and these were used to build structure models of the ligases in the open and closed conformations, respectively. Disulfide prediction used the DiANNA web server (http://clavius.bc.edu/~clotelab/DiANNA/)^22^. Leader sequences were predicted and removed prior to submission.

### Recombinant expression and purification of minimal ATP-dependent DNA ligase from Burkholderia pseudomallei (Bsp-lig)

The coding sequence for the ATP-dependent DNA ligase of *Burkholderia pseudomallei* strain BES (Bsp-lig), WP_050042554 was ordered from the Thermofisher GeneArt service with codon optimization for *E. coli*, and included an N-terminal hexa-histidine tag (His-tag) and TEV cleavage site at the N terminus. *bsp-lig* was sub-cloned into the pDEST 17 vector using the Gateway system (Thermo Fisher Scientific) and expressed as described previously for the Lig E protein from *Aliivibrio salmonicida*^17^. Briefly, an overnight culture of transformed BL21(DE3)Star cells were inoculated into Terrific Broth (TB) medium and grown at 37 °C until an OD_600_ of 0.3 was reached. Hereafter the temperature was decreased to 15 °C and protein expression was induced using 0.1 mM of IPTG. Cells were harvested after 18 h. Bsp-lig was purified to homogeneity using a two-step IMAC protocol as described^17^. Initial immobilized metal affinity chromatography (IMAC) purification was used to obtain His-tagged protein. Cells were lysed using a French press at 18 psi in lysis buffer (50 mM Tris pH 8.0, 750 mM NaCl, 10 mM MgCl_2_, 5% glycerol) and clarified cell lysate was incubated overnight in the presence of 0.l mM ATP at 4 °C. Cell lysate was loaded onto a 5 ml HisTrap HP column (Sigma-Aldrich) using binding buffer A (50 mM Tris pH 8.0, 750 mM NaCl, 10 mM imidazole, 5% glycerol) and washed with 10–15 column volumes of buffer A to remove *E. coli* contaminants. His-tagged protein was eluted on a linear gradient of 0–100% elution buffer B (50 mM Tris pH 8.0, 750 mM NaCl, 500 mM imidazole, 5% glycerol) and fractions containing His-Bsp-lig (approximately 60–80% B; 300–400 mM imidazole) were buffer exchanged into TEV cleavage buffer C (50 mM Tris pH 8.0, 200 mM NaCl, 5% glycerol, 1 mM DTT) using a HiPrep 26/10 (Sigma-Aldrich). His-Bsp-lig was then digested overnight with His tagged TEV protease^33^ at 4 °C. This cleaved protein was subjected to a reverse IMAC step in buffer C to obtain His-tag-free DNA ligase in the flow-through fraction. The follow-through fraction was up-concentrated to a volume less than 5 mL using Amicon Ultra centrifugal filter units Ultra-15, MWCO 10 kDa (Amicon). Up concentrated Bsp-lig was polished by gel filtration on a HiLoad 16/600 Superdex 200 column in buffer C before use in assays, or crystallization trials.

### DNA ligase activity assays

Ligase activity was measured by molecular beacon assay as previously described^16,34^. Unless otherwise stated, the reaction conditions were 300 nM substrate, 0.1 mM ATP, 10 mM MgCl_2_, 1.0 mM 1,4-dithiothreitol (DTT), 100 mM NaCl, 50 mM Tris pH 8.0 at 30 °C.

Ligase activity with double- and single-stranded breaks were measured by denaturing urea-PAGE of fluorescently-labelled DNA duplexes as described previously^35^; (briefly, 80 nM substrate, 0.1 mM ATP, 10 mM MgCl_2_, 1.0 mM 1,4-Dithiothreitol (DTT), 100 mM NaCl, 50 mM Tris pH 8.0) and with the following assay conditions: nicked substrate 15 min at 25 °C; cohesive overhang substrate 2 h 25 °C; mismatch substrate 2 h 15 °C, blunt and overhang substrate 2 h and 18 h 15 °C. DNA oligos used to assemble substrates are given in Supplementary Tables 4 and 5.

### Crystallization and structure determination

The DNA substrate for co-crystallization was assembled from HPLC-purified oligos purchased from IDT 5_P-strand: (Phos) CAC TAT CGG AA; Complementary-strand: TTC CGA TAG TGG GGT CGC AAT; 3_OH-strand: ATT GCG ACC where the underlined nucleotide is a modified 2-*O*-methylcytidine. Oligomers were resuspended at 9 mM in annealing buffer (50 mM Tris pH 8.0, 50 mM NaCl, 1 mM EDTA), mixed 1:1:1 to give a final duplex concentration of 3 mM and incubated at 85 °C before cooling overnight. Bsp-lig (414 µM) was incubated with 1.2 molar equivalents of nicked duplex and 5 mM additional EDTA for up to 1 h on ice to form the protein-DNA complex. Crystals with a thin plate morphology were grown by hanging drop diffusion method at 4 °C from 26% PEG 3350, 100 mM Bis–Tris pH 5.5. Crystals were cryoprotected in 26% PEG 3350, 100 mM Bis–Tris pH 5.5, 12% ethyleneglycol and flash frozen in liquid nitrogen. Diffraction data to 2.45 Å was measured at BL14.1, BESSY II, Berlin. Data was integrated, scaled and truncated in XDS, XSCALE^36^ and AIMLESS^37^. The complex structure was solved by molecular replacement using Phaser-MR^38^ with Chlorella virus DNA–protein complex (PDB ID: 2Q2T) and Psy-Lig enzyme-adenylate (PDB ID: 4D05) as search models, and further refined in Phenix.refine^39^ and manually built in COOT^40^. Data collection and statistics are listed in Table 5 and the structure was deposited to the Protein Data Bank with the identifier 7OBN.

**Table 5:** Data collection and refinement statistics for the Bsp-lig DNA crystal structure PDB 7OBN.n.

**Data collection**	
Wavelength (Å)	0.9184
Beamline	Bessy BL14.1 (18.08.17)
Resolution range (Å)	48.31–2.45 (2.55–2.45)
Space group	C2
Unit cell a, b, c (Å), α, β, γ (°)	125.47, 45.55, 111.46, 90, 103.94, 90
Total no. of reflections	80,324 (5873)
Unique no. of reflections	22,036 (2031)
Multiplicity	3.6 (2.9)
Completeness (%)	96.4 (79.4)
Mean I/sigma(I)	7.9 (0.9)
Wilson B-factor (Å^2^)	56.09
R-merge	0.084 (0.957)
R-meas	0.111 (1.303)
R-pim	0.056 (0.724)
CC1/2	0.990 (0.578)
**Refinement**	
Resolution range (Å)	24.62–2.45 (2.54–2.45)
Reflections used in refinement	21,969 (1775)
Reflections used for R-free	2021 (165)
R-work	0.2465 (0.3543)
R-free	0.2739 (0.3999)
Number of non-hydrogen atoms	3304
Macromolecules	3218
Ligands	26
Solvent	60
Protein residues	299
RMS (bonds) (Å)	0.030
RMS (angles) (°)	1.50
Ramachandran favored (%)	85.57
Ramachandran allowed (%)	12.03
Ramachandran outliers (%)	2.41
Rotamer outliers (%)	1.63
Clashscore	22.02
Average B-factor (Å^2^)	72.63
Macromolecule (Å^2^)	72.94
ligands (Å^2^)	65.56
Solvent	59.19
Number of TLS groups	8

Statistics for the highest-resolution shell are shown in parentheses.

## Supplementary Information

Supplementary Information.

## Data Availability

Atomic coordinates and structure factors for the reported crystal structures have been deposited with the Protein Data bank under accession numbers 7obn.
