# Supplementary material for: Bacteriophage origin of some minimal ATP-dependent DNA ligases: a new structure from Burkholderia pseudomallei with striking similarity to Chlorella virus ligase
Source: Sci Rep. 2021 Sep 21;11:18693. doi: 10.1038/s41598-021-98155-w (PMC8455567; doi:10.1038/s41598-021-98155-w)
Supplement: Supplementary file 1 — Supplementary Information. [file 41598_2021_98155_MOESM1_ESM.pdf]

Supplementary material: Bacteriophage origin of some minimal ATP-dependent DNA ligases: a new structure from *Burkholderia pseudomallei* with striking similarity to Chlorella virus ligase

Jolyn Pan[1], Kjersti Lian[2], Aili Sarre[2], Hanna-Kirsti S. Leiros[2] and Adele Williamson[1, 2]*
[1] School of Science, University of Waikato, Hamilton 3240, New Zealand
[2] Department of Chemistry, UiT The Arctic University of Norway, Tromsø, N-9037, Norway
* To whom correspondence should be addressed. Email: adelew@waikato.ac.nz

**Bacterium**	**ATP-dependent DNA ligase Uniprot identifier**
Actinobacillus indolicus.	A0A4P7CEM4
Actinobacillus ureae ATCC 25976.	E8KK51
Alteromonas lipolytica.	A0A1E8FI42
Alteromonas macleodii (strain Balearic Sea AD45).	K0EC25
Alteromonas mediterranea.	S5BYS1
Arcobacter anaerophilus.	A0A4Q0Y3K8
Campylobacter coli.	A0A3I7XBS7
Campylobacter concisus ATCC 51562.	U2F5T9
Campylobacter concisus.	A0A1X4MGH7
Campylobacter curvus (strain 525.92).	A7GWV3
Campylobacter gracilis RM3268.	C8PLL2
Campylobacter iguaniorum.	A0A076FAH4
Campylobacter jejuni subsp. Doylei (strain ATCC BAA-1458).	A7H619
Campylobacter jejuni subsp. Jejuni serotype O:23	A0A0H3PA79
Campylobacter lari (strain RM2100).	B9KEB9
Campylobacter lari NCTC 11845.	A0A0A8HT75
Campylobacter lari RM16712.	A0A0A8I9J1
Campylobacter novaezeelandiae.	A0A4Q9JVC0
Campylobacter peloridis LMG 23910.	A0A0A8GTI2
Campylobacter pinnipediorum subsp. Pinnipediorum.	A0A1S6TMG5
Campylobacter rectus RM3267.	B9D382
Campylobacter showae RM3277.	C6RIM0
Campylobacter sp. 113.	A0A2A4L8C3
Campylobacter sp. P255.	A0A420VLA9
Campylobacter sp. RM16704.	A0A0A8GVK8
Campylobacter sp. US12a.	A0A5R1LGW9
Campylobacter sp. US25a.	A0A5E9WQ41
Campylobacter sp. US33a.	A0A4Y8BSD2
Campylobacter subantarcticus.	A0A0A8HAL8
Candidatus Magnetomorum sp. HK-1.	A0A0M9ECM2
Colwellia psychrerythraea.	A0A099KNG4

Eikenella corrodens CC92I.	V7IE47
Ferrimonas sediminum.	A0A1G8TBD7
Haemophilus haemolyticus M19501.	F9GMF5
Haemophilus haemolyticus M21127.	F9GUM5
Haemophilus influenzae (strain 86-028NP).	Q4QLB6
Haemophilus influenzae (strain ATCC 51907 / DSM 11121 / KW20 /Rd).	P44121
Haemophilus influenzae (strain NTHi 3655).	A0A0H3PM16
Haemophilus influenzae F3031.	E7A588
Helicobacter cinaedi ATCC BAA-847.	I7GTY9
Helicobacter jaachi.	A0A4U8TCZ2
Helicobacter magdeburgensis.	A0A4U8SY23
Helicobacter sp. MIT 11-5569.	A0A4U8SFL6
Hydrogenovibrio thermophilus.	A0A410H4C7
Marinobacter salarius.	A0A1W6K568
Marinobacterium aestuarii.	A0A1A9EW76
Muribacter muris.	A0A4Y9K0J0
Neisseria animaloris.	A0A1X3CN72
Neisseria cinerea ATCC 14685.	D0W4A0
Neisseria longate subsp. Glycolytica ATCC 29315.	A0A0B5CFP4
Neisseria gonorrhoeae (strain ATCC 700825 / FA 1090).	Q5F595
Neisseria gonorrhoeae DGI2.	D6HB39
Neisseria lactamica (strain 020-06).	E4ZAZ1
Neisseria lactamica.	A0A1V0DRH7
Neisseria meningitidis (strain alpha14).	C6S4M8
Neisseria meningitidis alpha153.	C6SEQ5
Neisseria meningitidis M0579.	A0A0G4BXU0
Neisseria meningitidis serogroup B / serotype 15 (strain H44/76).	E6MZN5
Neisseria meningitidis serogroup B.	A0A0H5QXB9
Neisseria meningitidis serogroup C / serotype 2a (strain ATCC 700532).	A1KWC7
Neisseria mucosa ATCC 25996.	D2ZXK0
Neisseria sicca ATCC 29256.	C6M1S0
Neisseria zalophi.	A0A5J6PWP0
Oceanisphaera arctica.	A0A2P5TL84
Oceanisphaera psychrotolerans.	A0A1J4QHW1
Oleispira antarctica.	A0A1Y5HF29
Pasteurella bettyae CCUG 2042.	I3DEQ0
Pasteurella testudinis DSM 23072.	A0A1W1V1D9
Psychromonas sp. SP041.	A0A0A6YVN6
Rhodoferax ferrireducens.	A0A1W9KTK4
Rodentibacter mrazii.	A0A1V3IFV1
Rodentibacter pneumotropicus.	A0A1V3K0F6

Rodentibacter ratti.	A0A1V3L9A6
Shewanella amazonensis (strain ATCC BAA-1098).	A1S744
Shewanella livingstonensis.	A0A3G8LVU4
Shewanella polaris.	A0A4Y5YFU0
Variovorax boronicumulans.	A0A250DKK5
Vibrio aerogenes.	A0A1M5Z756
Vibrio alginolyticus.	A0A0L7YSC1
Vibrio cholerae RC385.	D7H906
Vibrio fluvialis.	A0A0X8LM10
Vibrio metoecus.	A0A0Q0TQ58
Vibrio quintilis.	A0A1M7YUC7

Supplementary table 1. UniProt Identifiers for ATP-dependent DNA ligases used to construct the phylogenetic tree in Figure 2

**Organism**	**ATP-dependent DNA ligase NCBI identifier**
Burkholderia pseudomallei.	WP_050042554.1
Burkholderia ubonensis.	WP_059926806.1
Burkholderia diffusa.	WP_059466471.1
Burkholderia cenocepacia.	WP_077056196.1
Burkholderia sp. AU17325.	WP_089452168.1
Burkholderia territorii.	KWH13560.1
Burkholderia cepacia.	WP_060050868.1
Burkholderia lata.	WP_069252215.1
Burkholderia vietnamiensis.	WP_059895115.1
Burkholderia thailandensis.	WP_156530277.1
Ralstonia solanacearum.	WP_052485970.1
Ralstonia solanacearum FJAT-1458.	APF87578.1
Pseudomonas phage JG054.	ARB11192.1
Stenotrophomonas phage DLP4.	ATS92210.1
Pseudomonas phage PaMx25.	YP_009603603.1
Pseudomonas phage JG012.	ARB11117.1
Pseudomonas phage NP1.	YP_009285862.1
Ralstonia phage RSJ2.	YP_009216555.1
Xanthomonas phage Xoo-sp2.	ANT45243.1
Pararhodospirillum photometricum.	WP_014415654.1
MULTISPECIES: *Neisseria.*	WP_097784573.1
Xanthomonas campestris.	WP_155400850.1
Verrucomicrobiales bacterium.	HBJ83482.1
Desulfurellales bacterium.	TXH47257.1
Aeromonas sobria.	WP_148612331.1
Synechococcus phage S-CRM01.	YP_004508627.1
Pseudoalteromonas phage PH1.	YP_009321550.1
Synechococcus sp. WH 8016.	WP_006852967.1

Supplementary table 2. Identifiers for ATP-dependent DNA ligases used to construct the phylogenetic tree in Figure 5.

**Organism**	**Name**	**Directio n**	**locus_tag**	**protein_id**
V. cholera	methyl-accepting chemotaxis protein	forward	MS6_1320	BAP02975.1
	cell wall-associated hydrolase	reverse	MS6_1321	BAP02976.1
	type IIA topoisomerase, B subunit	forward	MS6_1322	BAP02977.1
	ribulosamine/erythrulosamine 3-kinase potentially involved in protein deglycation	reverse	MS6_1323	BAP02978.1
	hypothetical protein	reverse	MS6_1324	BAP02979.1
	multidrug and toxin extrusion MATE family effluxpump YdhE/NorM	reverse	MS6_1325	BAP02980.1
	hypothetical protein	reverse	MS6_1326	BAP02981.1
	**DNA ligase**	**forward**	**MS6_1327**	**BAP02982.1**
	TPR domain protein, putative component of TonB system	forward	MS6_1328	BAP02983.1
	ferric siderophore transport system, periplasmic binding protein TonB	forward	MS6_1329	BAP02984.1
	biopolymer transport protein ExbD/TolR	forward	MS6_1330	BAP02985.1
	ferric siderophore transport system, biopolymer transport protein ExbB	forward	MS6_1331	BAP02986.1
	MotA/TolQ/ExbB proton channel family protein	forward	MS6_1332	BAP02987.1
	TonB system biopolymer transport component	forward	MS6_1333	BAP02988.1
	glycerol-3-phosphate ABC transporter, periplasmic glycerol-3-phosphate-binding protein	reverse	MS6_1334	BAP02989.1
	glycerol-3-phosphate ABC transporter, permease protein UgpA	reverse	MS6_1335	BAP02990.1
N. meningitides	phosphomethylpyrimidine synthase ThiC	forward	CCD84_11490	QEN75775.1
	tRNA-Met	reverse	CCD84_11485	
	hypothetical protein	reverse	CCD84_11480	
	NUDIX hydrolase	reverse	CCD84_11475	QEN75774.1
	ABC transporter ATP-binding protein	reverse	CCD84_11470	QEN75773.1
	hypothetical protein	reverse	CCD84_11465	QEN75772.1
	phosphoenolpyruvate—protein phosphotransferase	forward	CCD84_11460	QEN75771.1
	phosphocarrier protein HPr	forward	CCD84_11455	QEN75770.1
	PTS mannose transporter subunit IIA	forward	CCD84_11450	QEN75769.1
	hypoxanthine-guanine phosphoribosyltransferase	forward	CCD84_11445	QEN75768.1
	**ATP-dependent DNA ligase**	**forward**	**CCD84_11440**	**QEN75767.1**
	MBL fold hydrolase	reverse	CCD84_11435	QEN75766.1
	DNA recombination protein RmuC	forward	CCD84_11430	QEN75765.1
	cytochrome c1	forward	CCD84_11425	QEN75764.1
	cytochrome b	forward	CCD84_11420	QEN75763.1
	ubiquinol-cytochrome c reductase iron-sulfur subunit	forward	CCD84_11415	QEN75762.1

	Nif3-like dinuclear metal center hexameric protein	forward	CCD84_11410	QEN75761.1
H. influenzae	branched-chain amino acid aminotransferase	reverse	NF38_08205	AIT68164.1
	uvrD	reverse	NF38_08210	AIT68362.1
	peptide ABC transporter permease	reverse	NF38_08215	AIT68165.1
	peptide transporter	reverse	NF38_08220	AIT68166.1
	dppD	reverse	NF38_08225	AIT68167.1
	dppF	reverse	NF38_08230	AIT68168.1
	**DNA ligase**	**forward**	**NF38_08235**	**AIT68169.1**
	gmhA	reverse	NF38_08240	AIT68170.1
	arginine ABC transporter ATP-binding protein	reverse	NF38_08245	AIT68171.1
	arginine ABC transporter substrate-binding protein	reverse	NF38_08250	AIT68172.1
	ABC transporter	reverse	NF38_08255	AIT68173.1
	artM	reverse	NF38_08260	AIT68174.1
	hypothetical protein	reverse	NF38_08265	AIT68363.1
	protein opa	forward	NF38_08270	AIT68175.1
	sprT	forward	NF38_08275	AIT68176.1
	S-adenosylmethionine synthetase	forward	NF38_08280	AIT68177.1
C. jejuni	putative integral membrane protein	reverse	Cj1660	CAL35756.1
	possible ABC transport system permease	reverse	Cj1661	CAL35757.1
	putative integral membrane protein	reverse	Cj1662	CAL35758.1
	putative ABC transport system ATP-binding protein	reverse	Cj1663	CAL35759.1
	putative periplasmic thioredoxin	reverse	Cj1664	CAL35760.1
	putative lipoprotein thioredoxin	reverse	Cj1665	CAL35761.1
	putative periplasmic protein	forward	Cj1666c	CAL35762.1
	RepA protein homolog	forward	Cj1667c	CAL35763.1
	putative periplasmic protein	forward	Cj1668c	CAL35764.1
	**putative ATP-dependent DNA ligase**	**forward**	**Cj1669c**	**CAL35765.1**
	cgpA	forward	Cj1670c	CAL35766.1
	hypothetical protein Cj1671c	forward	Cj1671c	CAL35767.1
	eno	forward	Cj1672c	CAL35768.1
	recA	forward	Cj1673c	CAL35769.1
	conserved hypothetical protein Cj1674	reverse	Cj1674	CAL35770.1
	fliQ	reverse	Cj1675	CAL35771.1
	murB	reverse	Cj1676	CAL35772.1
	putative lipoprotein	reverse	Cj1677	CAL35773.1

Supplementary table 3. Genes for region surrounding Lig E from pathogen genomes used in analysis

**Oligo name**	**Sequence 5'-3'**	**Modifications**
Oligo_1 (Nick)	AGGCCATGGCTGATATCGCA	5' 6-FAM
Oligo_2	TAGGCATTCGAGCTCCGTCG	5' Phosphate
Oligo_3(nick)	CGACGGAGCTCGAATGCCTATGCGATATCAGCCATGGCCT	
Oligo_4 (OH)	ATATCAGCCATGGCCT	5' Phosphate
Oligo_5 (OH)	CGACGGAGCTCGAATGCCTATGCG	
Oligo_6 mis	CGACGGAGCTCGAATGCCTACGCGATATCAGCCATGGCCT	

Oligo_7	TGCGATATCAGCCATGGCCT	
Oligo_8	CGACGGAGCTCGAATGCCTA	5’ Phosphate
Oligo_9	CGACGGAGCTCGAATGCCTAGTGCGATATCAGCCATGGCCT	

Supplementary table 4. DNA oligomers used to assemble assay substrates

**Substrate**	**Oligo combination**
Nick	Oligo_1 + 2 + 3
Cohesive	Oligo_1 + 2 + 4 + 5
Mismatch	Oligo_1 + 2 + 6
Blunt	Oligo_1+ 2 + 7
Gapped	Oligo_1 + 2 + 9

Supplementary table 5. Combinations of DNA oligos used to assemble assay substrates

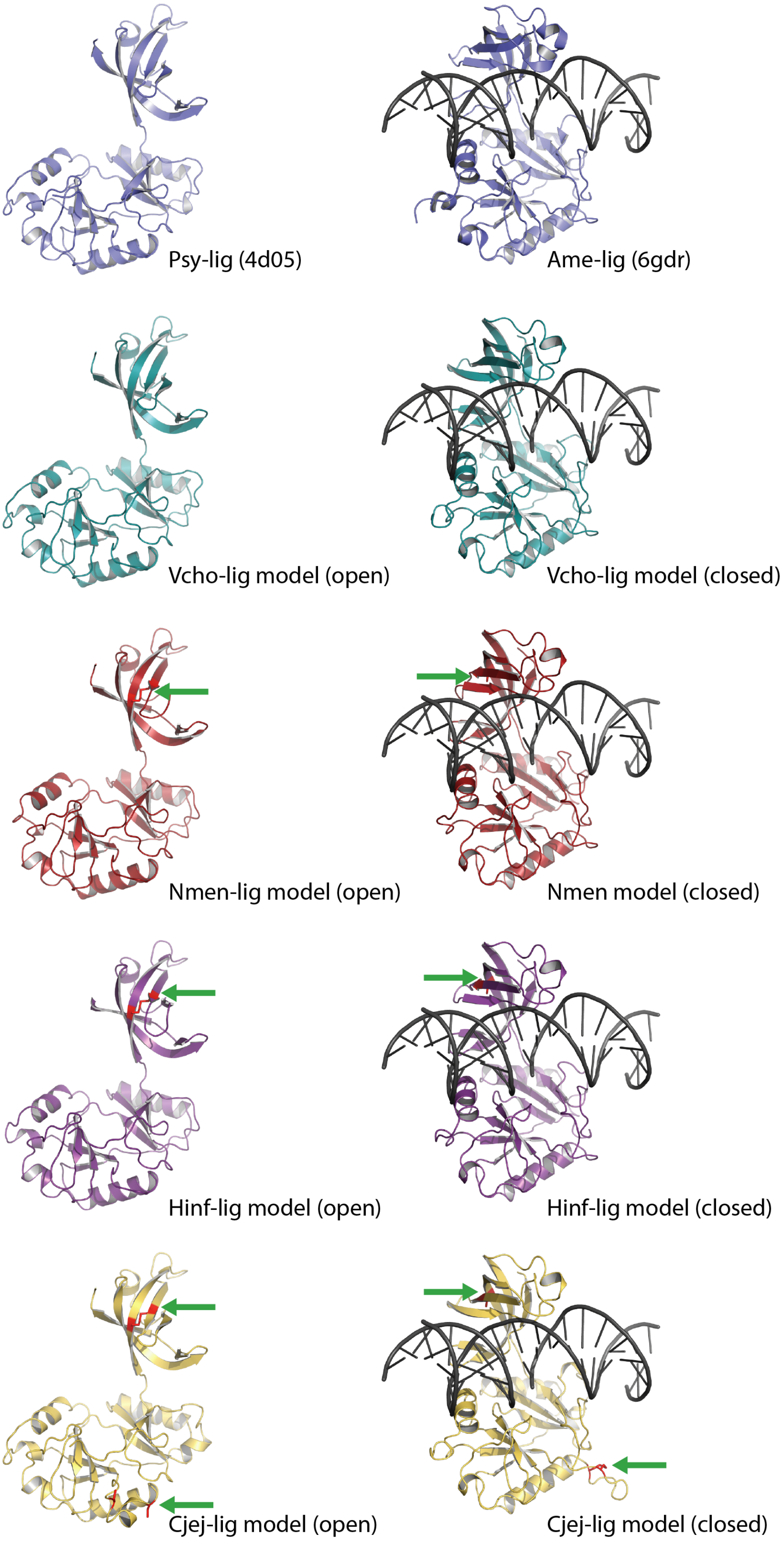
Psy-lig (4d05)
Ame-lig (6gdr)
Vcho-lig model (open)
Vcho-lig model (closed)
Nmen-lig model (open)
Nmen model (closed)
Hinf-lig model (open)
Hinf-lig model (closed)
Cjej-lig model (open)
Cjej-lig model (closed)

Supplementary figure 1. Homology models of Lig E from various pathogens in both the DNA-free (open) and DNA-bound (closed) conformations. Cysteine residues are colored red and their positions indicated with green arrows.

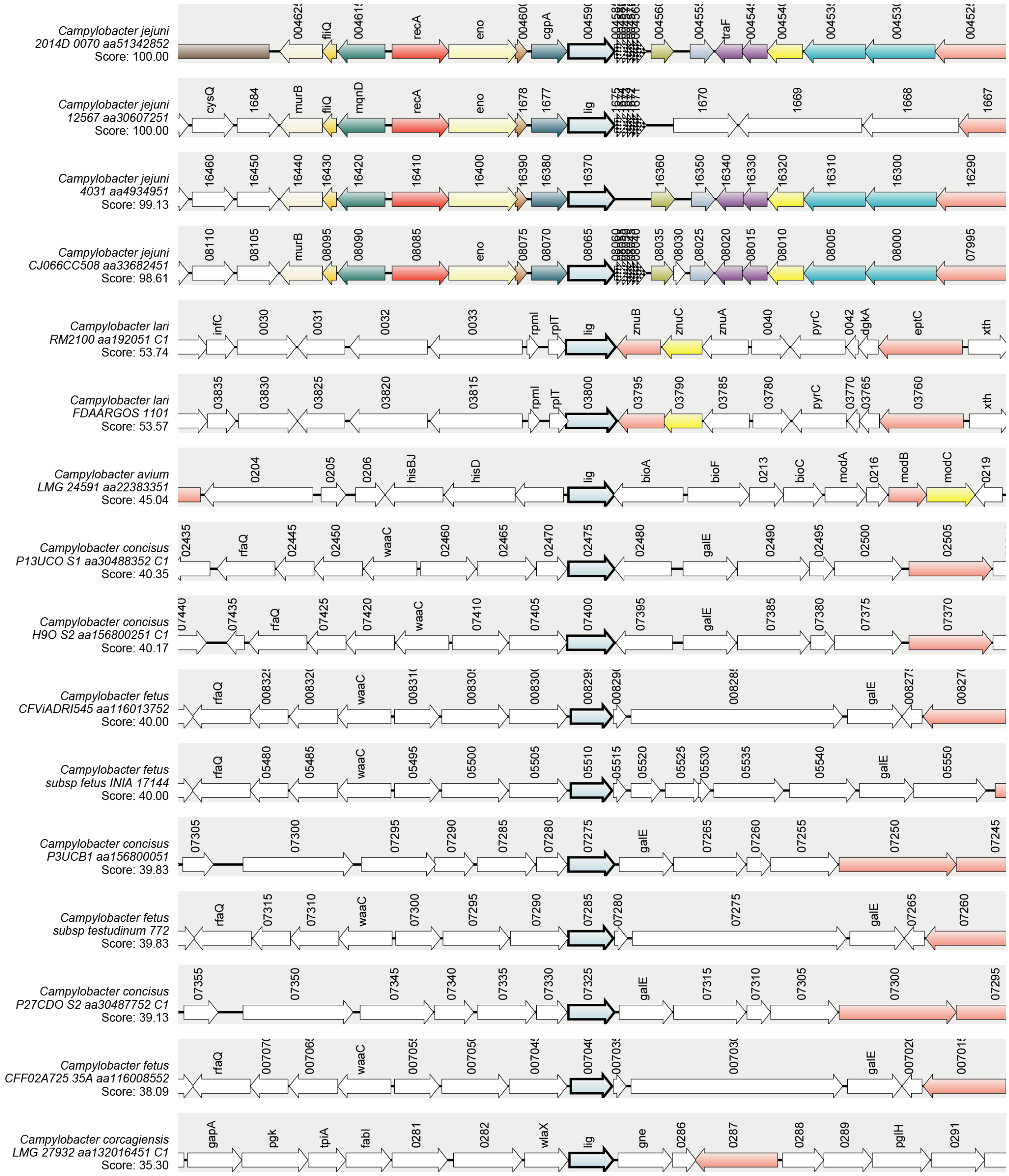

Supplementary figure 2. Synteny analysis of the Lig E region of Campylobacter genomes

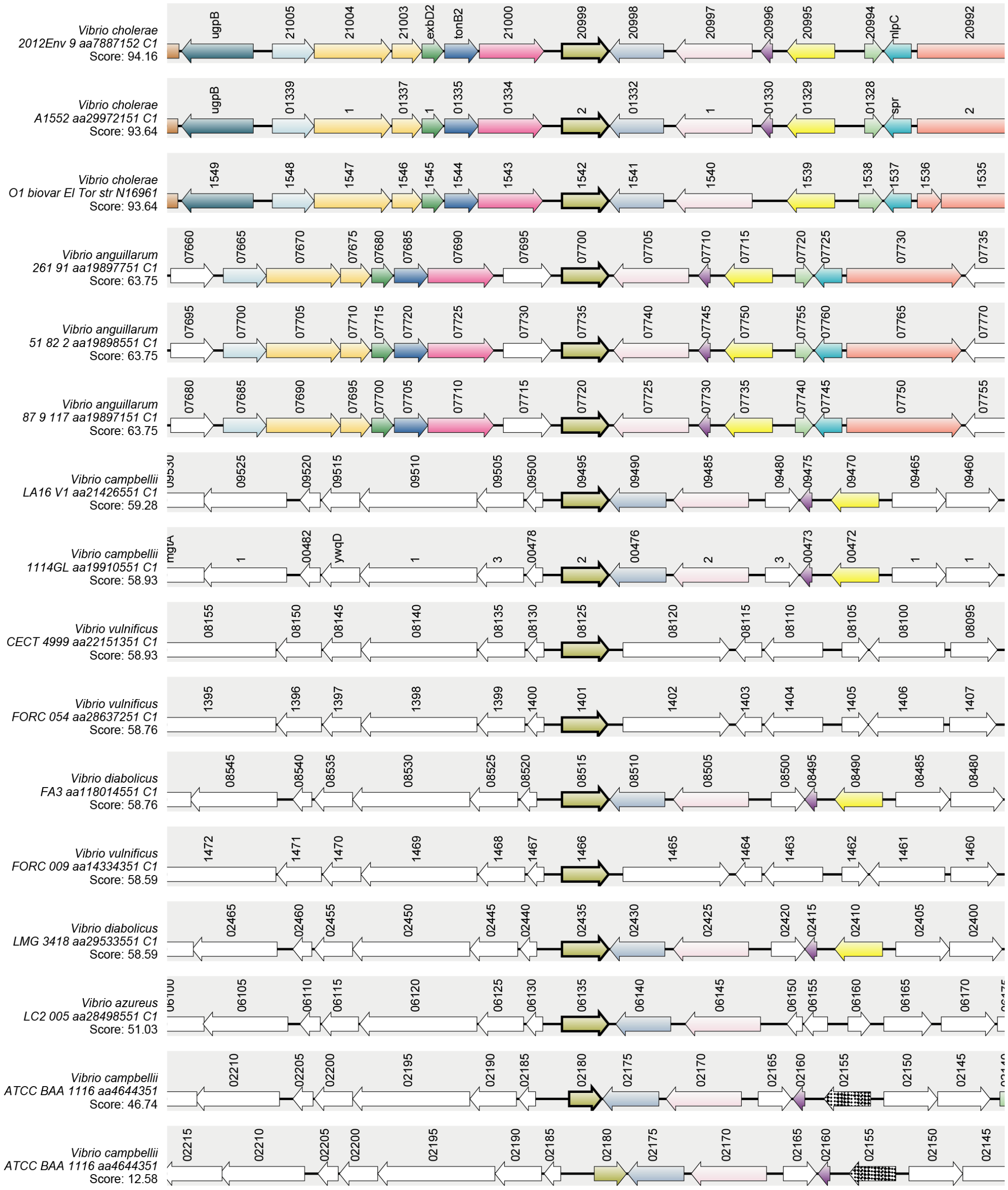

Supplementary figure 3. Synteny analysis of the Lig E region of Vibrio genomes

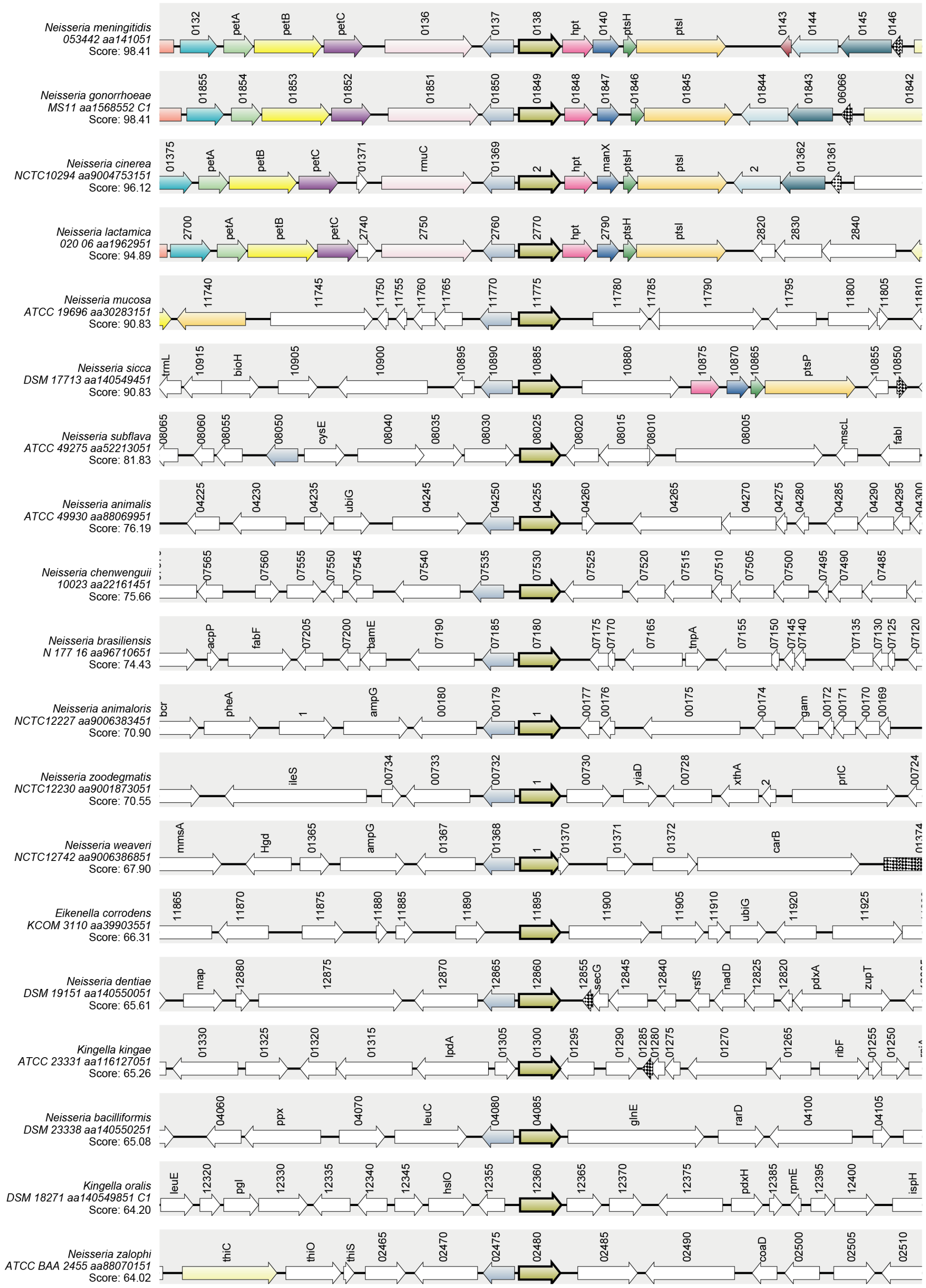

Supplementary figure 4. Synteny analysis of the Lig E region of Neisseria genomes

Genomic contexts

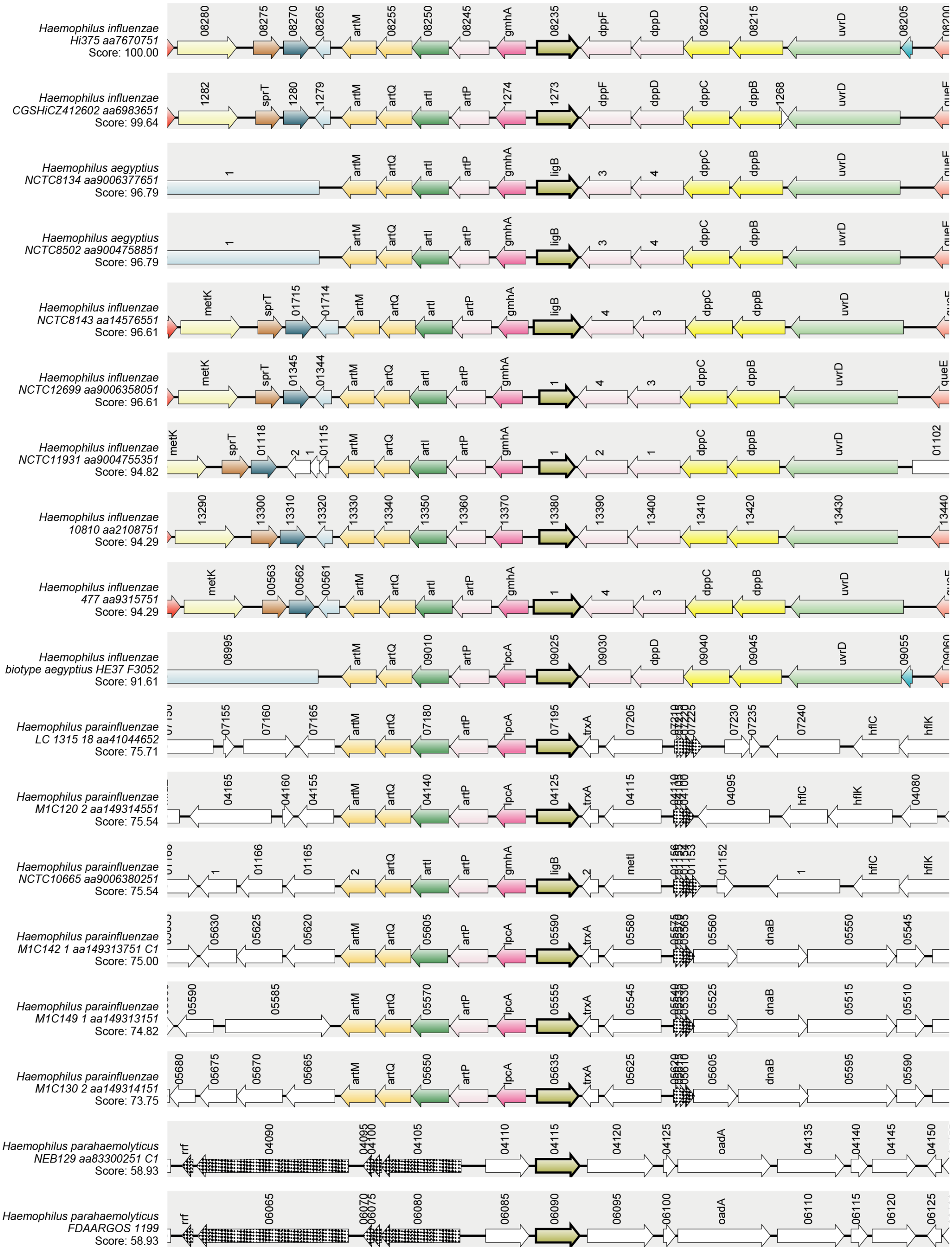

Supplementary figure 5. Synteny analysis of the Lig E region of Haemophilus genomes

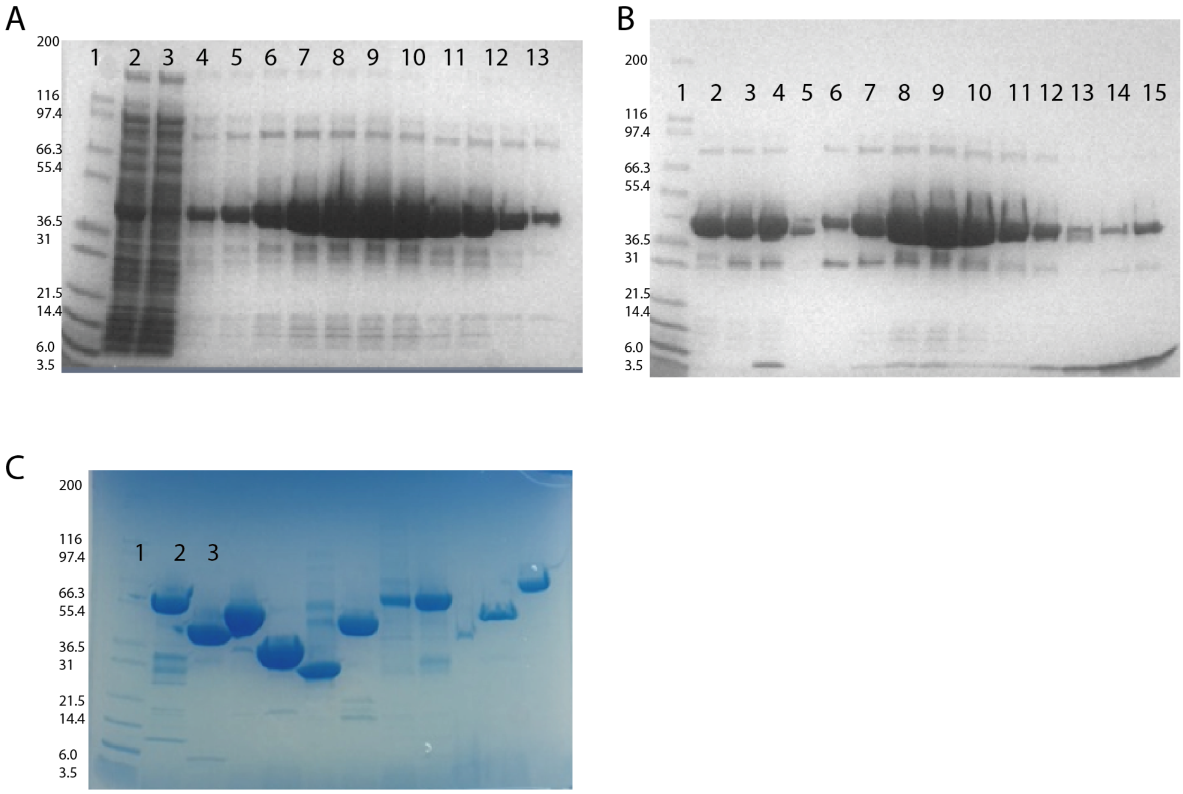

Supplementary figure 6. SDS-PAGE gels of Bsp-lig purification lane 1 is protein standard Mark12 for all gels. A) First IMAC showing crude soluble (lane 1) flow-through (lane 2) and elution fractions between 300-400 mM imidazole (lanes 4-13). B) TEV cleavage and second (reverse) IMAC showing pooled fractions from the desalt step (lane 2) pooled factions + TEV at time =0 (lane 3) and after overnight incubation (lane 4), pooled flow through fractions from the reverse IMAC (lane 5) and elution fractions of the reverse IMAC (i.e. uncleaved Bsp-lig, TEV and TEV). C) Pooled and up-concentrated Bsp-lig after gel filtration (lane 3). Lane 2 and lanes on the right of the image (un-labeled) are unrelated protein samples from another project.

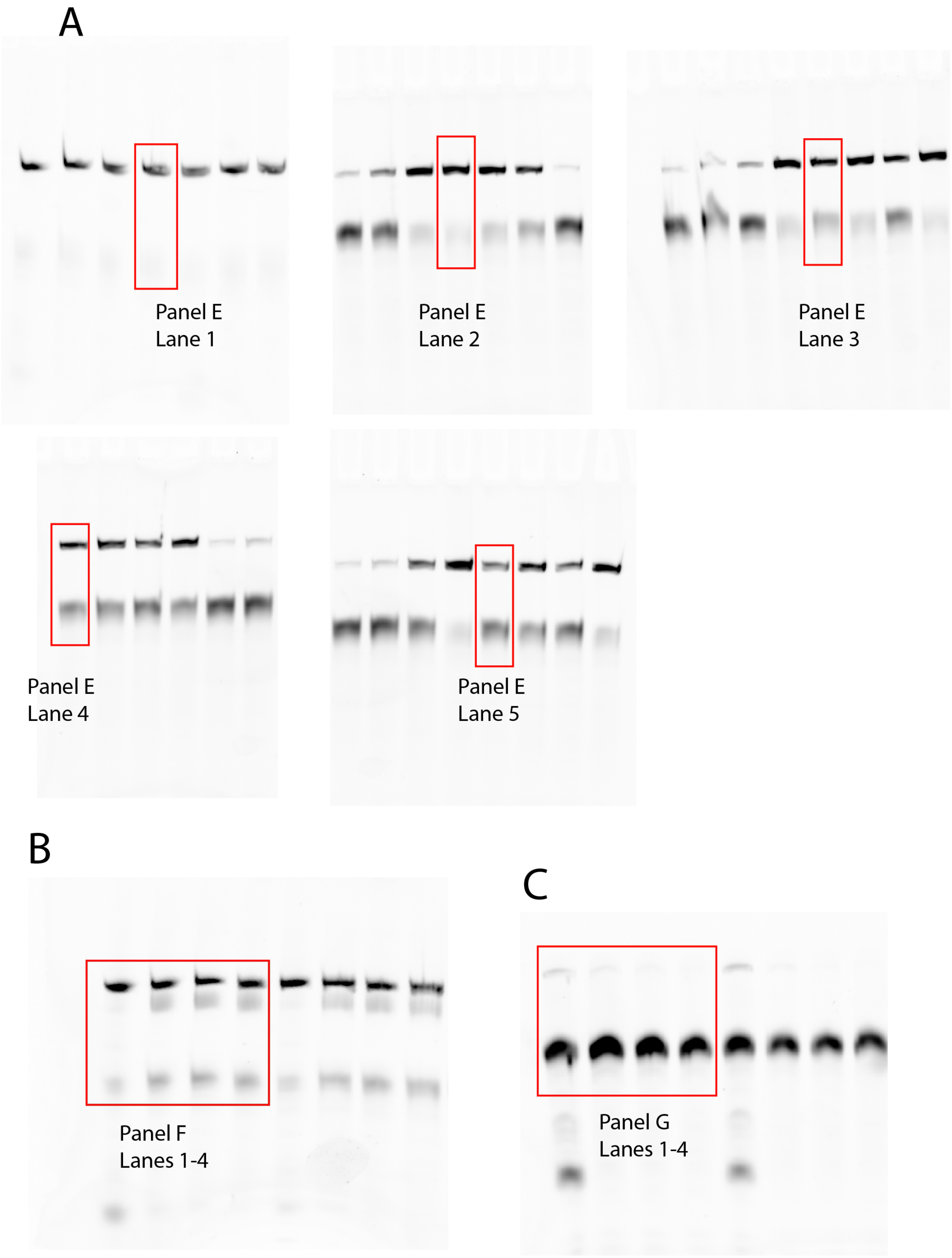

Supplementary figure 7. Uncropped gels from figure 3. A) Bsp-lig activity with nicked substrate (Figure 3 panel E). These samples were run non-sequentially across multiple gels interspersed with samples from another project; lanes containing Bsp-lig are shown in red boxes. B) Bsp- lig activity with cohesive-end substrate (Figure 3 panel F). Region used in the crop is indicated with a red box. Samples to the right are duplicates. C) Bsp-lig activity with mismatch substrate (Figure 3 panel G). Region used in the crop is indicated with a red box. Samples to the right are duplicates. In both B and C the lower molecular weight band is indicative of trace nuclease contamination with higher protein concentrations.

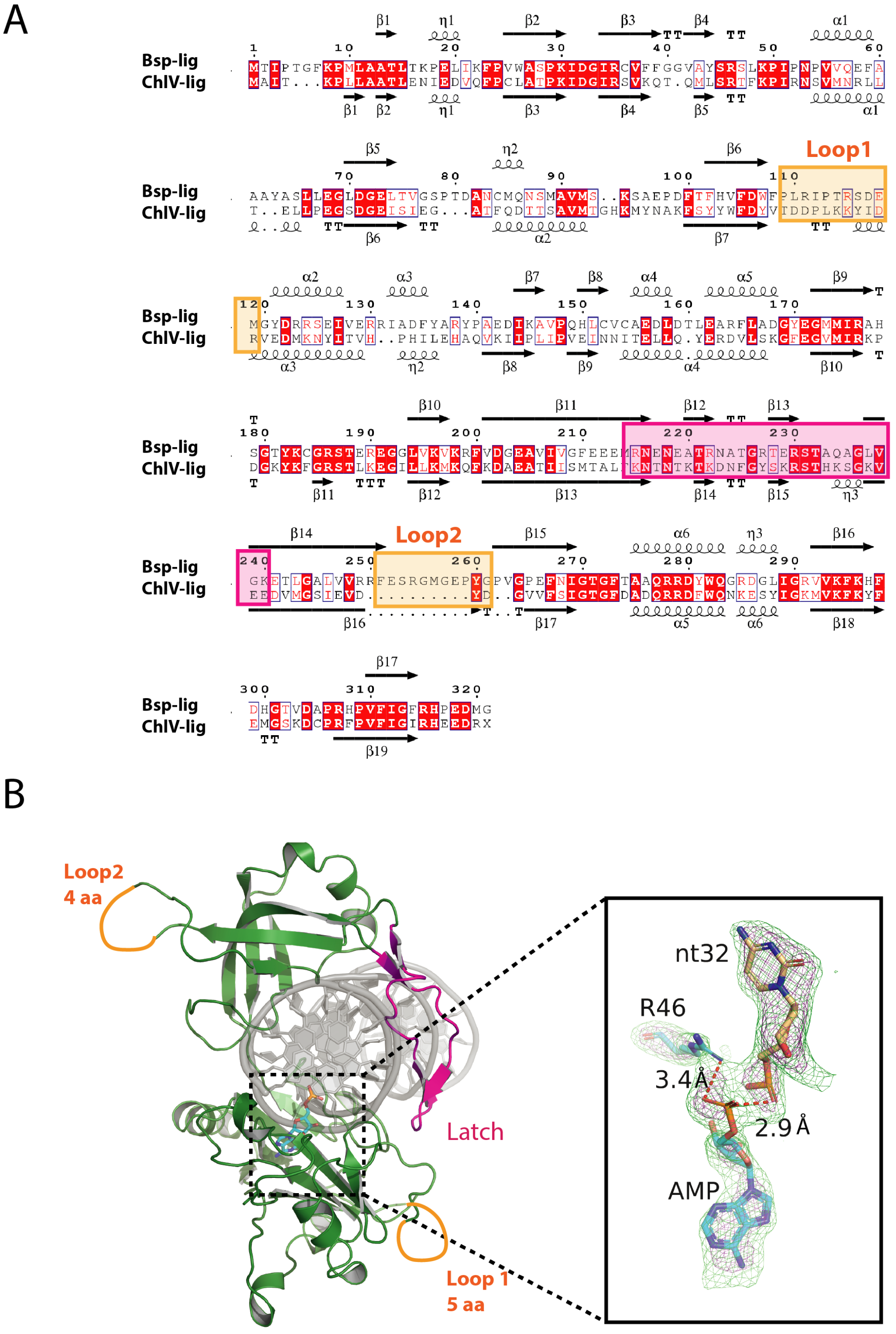

Supplementary figure 8. A) Sequence alignment of Bsp-lig and ChlV-lig showing secondary structure alignments from the current Bsp-lig structure (7obn) and ChlV-lig (2q2t) above and below respectively. Loop regions of Bsp-lig are highlighted in yellow, latch region in magenta. B) Bsp-lig structure highlighting loop and latch regions. Inset shows the polder omit map of the spent Amp cofactor in the active site interacting with nt32 from the DNA substrate nick, and Arg46 in the Bsp-lig active site. Measured distances between atoms are indicated with dashed red lines.
